# Influence of Print Speed on the Mechanical Performance of 3D-Printed Bio-Polymer Polylactic Acid

**DOI:** 10.3390/ma18081765

**Published:** 2025-04-11

**Authors:** Ludwik Lorkowski, Katarzyna Wybrzak, Emila Brancewicz-Steinmetz, Jacek Świniarski, Jacek Sawicki

**Affiliations:** 1 Institute of Materials Science and Engineering, Faculty of Mechanical Engineering, Lodz University of Technology, Stefanowskiego 1/15, 90-924 Lodz, Poland; 242720@edu.p.lodz.pl (L.L.); katarzyna.wybrzak@dokt.p.lodz.pl (K.W.); emila.brancewicz-steinmetz@dokt.p.lodz.pl (E.B.-S.); 2 Department of Strength of Materials, Faculty of Mechanical Engineering, Lodz University of Technology, Stefanowskiego 1/15, 90-924 Lodz, Poland; jacek.swiniarski@p.lodz.pl

**Keywords:** print speed, bio-polymer, PLA, 3D printing

## Abstract

This study investigates the effect of 3D printing speed on the mechanical strength of parts produced with high-speed PLA. Samples were tested according to the ISO 527-1 standard, focusing on tensile strength. The results reveal that increasing the print speed from 30 mm/s to 500 mm/s reduces the mechanical strength of the samples, although the difference is minimal and does not affect the surface quality when the material is appropriately selected. Additionally, the orientation of the samples on the build plate had a significant impact on their strength, with samples printed along the Y-axis exhibiting better tensile performance. Ironing, which smooths the surface at the end of the print, improved the fracture surface consistency and tensile strength, regardless of the print speed. The improvement in tensile strength observed in ironed specimens can be attributed to improved bonding of the layers, reduced porosity, and a reduction in stress concentration points, which ultimately contributed to more uniform stress distribution and less risk of premature failure. Thermal camera images indicated no significant deviations in heat distribution, excluding this factor as a cause for inconsistent fracture points. This study concludes that higher printing speeds offer time and energy savings with minimal impact on mechanical properties, making them suitable for prototyping and decorative elements, although the effects of print speed and orientation should be considered for applications requiring higher strength.

## 1. Introduction

FFF (Fused Filament Fabrication) is a form of extrusion-based AM and a highly efficient 3D incremental prototyping method, providing many opportunities for innovation and advancement in the field [1,2]. The technology involves extruding molten material in a controlled manner through a heated nozzle.

The material is supplied as a filament with a standardised diameter (1.75 or 2.85 mm), wound on a spool [3].

The technology’s versatility is underscored by its support for a wide range of materials, sparking curiosity and inspiration for potential applications [4], making it possible to produce parts not only with complex geometries but also with unique strength properties, which are highly dependent on the printing parameters [5,6] PEEK (Polyetheretherketone) is a prime example of a material that can be 3D printed with unique properties, such as an exceptional strength-to-weight ratio, long-term chemical resistance, and a high melting point at 343 °C. These properties underscore the versatility of FFF technology and its potential applications in various fields.

The versatility of FFF’s application in various fields [7] accelerates the process of prototyping elements, which speeds up obtaining parts that meet the final design requirements. FFF printing is based on converting [8] 3D geometry [9] files into G-code [10] machine language, which allows us to transform digital 3D models into a set of layers [11] with modifiable parameters, which are then executed [10] by the 3D printer. This division of the 3D model into layers allows for the precise definition of the vertical direction’s rendering, which significantly influences the overall model’s details [12]. The layer height, a key parameter, plays a crucial role in defining the tensile strength [13], surface roughness [14], and the time required for printing the models [15]. Vertical resolution is related to the nozzle diameter, which remains in a close relationship and is taken into account when determining the parameters of printing [16,17] and the layer height increase.

PLA [18] (polylactic acid) material responds to the need for diversified sources of plastics produced through petrochemical processes [19], in the form of renewable components with biodegradable and biocompatible [20] properties.

As one of the most popular plastics, allowing for relatively easy 3D printing, it finds its popularity due to its low degree of shrinkage after extrusion [21], along with having the lowest extrusion temperature (190–230 °C) compared to other popular materials [22]. Due to the typical lack of high strength expectations from this material compared to technical materials [23] (which is a noticeable drawback), it is often used for printing at much higher speeds than technical materials, which frequently require low printing speeds due to post-extrusion shrinkage and the need for accurate and stable melting.

However, this allows the use of PLA to be extended to a level that technical materials cannot reach: the modification of pure PLA to improve its properties, providing it with an advantage over other materials in terms of ease of printing.

One such solution is a PLA material referred to as high-speed. It is characterised by a much more improved melt flow index [24] (MFI), due to the reduction in the viscosity and molecular weight of the thermoplastic. In addition to maintaining excellent printing stability in practice at high speeds, the material also has other features that increase its usefulness, such as higher mechanical strength and impact resistance, which increase its field of application in functional parts.

With the increasing popularity of printers capable of stable high-speed printing without excessive wear and tear on their components (this is mainly due to the use of linear guides [25], which provide vibration absorption and accurate positioning [26]), the emergence of high-speed materials means that the pace of development of FFF printers’ capabilities is not hindered, pushing the limits of these machines further. These 3D printers capable of high-speed printing must find a good balance between the fast movement of the extruder (which, as previously mentioned, is relatively easy to achieve with currently available components, without affecting the reliability of the equipment) and the proper material melting, ensuring that the material can reproduce the correct geometry of the part, maintain proper adhesion to the preceding layers, and be controlled by parameters exclusive to the extruder, such as feed speed, flow rate, and melting temperature. The main challenges are not focused on the extruder’s movement within the printer’s working area, but rather on the printer’s ability to consistently and precisely extrude material that maintains the expected properties, or those guaranteed by the material manufacturer. We are currently in an era of upgrading 3D printers to maximise their capabilities within the limitations imposed by both the hardware and emerging materials. The development of new materials requires the adjustment of printing parameters, which often leads to the modification of components within the printer. In turn, enhancing the hardware capabilities forces material manufacturers to adapt the material properties to the technical specifications of the printer.

In a very short time, desktop 3D printers have become significantly more user-friendly, limiting the user to simply loading the material into the feeding system (if the printer is equipped with one, such as the Bambu Lab AMS System) and starting the specific print. All processes, from model selection to the removal of the finished part from the print bed, are now handled by the printer based on manufacturer-provided code, which includes mechanisms like automatic nozzle cleaning from previously used materials, flow calibration, and nozzle calibration relative to the print bed. A full automation of processes that were once performed manually by the operator and often triggered by print failures has been achieved. These are now part of the printer’s automatic diagnostics before each print, to minimise defects such as clogged extruders.

The Bambu Lab A1 mini used in the tests for this paper represents the capabilities of rapid and precise part manufacturing in FFF technology while being one of the smallest available desktop printers. The miniaturisation of rapid prototyping equipment means that there is no need for overdesign to secure the structure, as the printer’s capabilities are maintained even in a lightweight and portable design. However, this smallest available printer has limitations regarding the use of certain materials. As long as the material used can maintain detail and quality at high throughput speeds without significant shrinkage that could lead to cracking and weakened layer adhesion, such as PLA, printers capable of achieving high speeds are not recommended for more demanding materials due to their open, non-chambered design.

Printing speed is not only one-sidedly positive when it allows us to prototype faster. It is one of the most critical parameters affecting the manufactured part’s final properties. This influence is evident in the cooling process of the extruded material, the pressing time that causes the top and bottom layers to merge, the material pull (which can affect the width of the extruded path) [27], and shrinkage. Finally, applying successive layers too quickly may not give us the correct dimensions, especially on the vertical axis, if the previous layers have not cooled and cured sufficiently. This would equate to uneven layer heights, resulting in uneven layer merging strength.

Understanding and adjusting the printing speed to match the material’s capabilities is crucial, empowering researchers and professionals to optimise their 3D printing processes.

Parameters such as layer height and speed are most important in determining the visual quality of 3D printing. The layer height right after the infill factor greatly impacts the strength of the printed part.

While some may argue that the infill pattern significantly impacts the strength of the printed part, research by Fernandez-Vicente et al. suggests that this influence is less than 5%. This finding provides a comprehensive understanding of the factors that affect the strength of the final product [28].

When searching for the optimal strength parameters, the selection of the printed part’s infill percentage is the most crucial aspect. This determines the movement of the nozzle in the XY-axis, the printing time, and the area filled with the material. The infill density can range from 0%, where the part is empty (shell geometry), to partially filled (1–99%) or solid (100%) [29].

To fully exploit the strength potential of the printed model, we can arrange the model on the platform when we know the filling pattern and, more precisely, the exact direction and order of filling the layers to ensure that the best strength properties will be determined in a particular direction of the model [30]. Research conducted by Suteja and Soesanti also raises the relevance of fill deposition speed [31]. This parameter is often used to reduce the printing time of parts that are not to be subjected to high loads, by increasing the printing speed of these parts of the layer. A prevalent practice is to have a higher print speed for the inner parts of the layers (inner walls and infill) while setting a low print speed for the outer layers to ensure the model’s visual quality and dimensional tolerance.

Printing speed is of great importance for the final product quality. It has been shown that the optimal printing parameters for the final geometry are, among others, 70 mm/s, but this also depends on cooling, nozzle temperature, and the filling or material [32,33]. In the work [34], it was shown that, for PLA, depending on many different factors, such as layer thickness as well as positioning on the platform, printing angle, and printing temperature, the ultimate tensile strength ranged between 16.41 MPa and 53.25 MPa. Young’s modulus reached a value between 2.22 GPa and 3.27 GPa.

The material used in our tests, Bambu PLA Basic, is described as being optimised for high-speed printing, making it an ideal match for printers capable of printing at high speeds. Certain processes, such as proper quality checks during production to ensure a deviation of 1.75 mm, are crucial for achieving repeatability and precision during printing and material feeding into the extruder. This ensures consistent material melting without temperature fluctuations, which are a common cause of nozzle clogs.

Certain thermoplastic materials with higher processing requirements necessitate the use of a heated chamber to maintain suitable environmental conditions for the printed part, as well as a slower cooling rate through airflow, requiring a heated chamber to prevent layer detachment due to increased surface stresses between successive layers, which are closely related to material shrinkage. The best example of the aforementioned limitations in high-speed printing is ABS. Being a petroleum-based polymer, it requires a higher printing temperature than PLA. It is used for manufacturing parts with higher mechanical strength, greater temperature resistance, and that are easier to process later on, such as smoothing with acetone vapours.

Other examples include PC, PEEK, and PA (nylon), which, due to their high melting temperatures and the need for extended processing time (resulting from their high viscosity) and temperature control, are not recommended for high-speed printing. To ensure the desired high-performance properties, these materials require excellent interlayer bonding, which can only occur if the material is melted for the right amount of time at high temperatures. Fast layer deposition causes temperature differences within the print, leading to distortions, cracks, or layer detachment from the print bed [35,36,37]. For ABS, the optimal printing speed (among other parameters) was determined to be 59.75 mm/s [38]. The poorest geometry reproduction was observed at a speed of 90 mm/s [39]. The literature also suggests that an increase in speed has a positive effect on the accuracy of some dimensions [40]. It has been shown that the printing speed also has a significant effect on surface roughness [41].

The printing speed also has a significant effect on strength properties. It has been shown that a lower speed allows for obtaining higher strength properties by obtaining better adhesion of the layers [42,43,44,45,46]. Depending on the printing speed and other parameters, even the type of printer, the tensile strength value may change many times. In the studies of Kamer M. et al. (2022), the tensile strength reached between 60 and 20 MPa [47]. This relationship was also demonstrated in research conducted by Miazio Ł. (2019), who showed that for printing speeds between 20 and 100 mm/s, the breaking force decreased from 740 N to 480 N, with a sharp decrease in these values occurring at a speed of 80 mm/s, which was caused by printing defects [48]. However, not all studies confirm this relationship because, in some, the tensile strength results are very similar for different speeds, and the weakest values are not always associated with the highest tested speed [49].

In addition to printing speed, another factor that has an equally significant impact on tensile strength is the orientation of the platform and the angle at which the samples were printed [50,51,52]. This is even indicated as the most critical printing parameter for tensile strength [53].

Print speed has such a significant impact on the mechanical properties of samples because of the correlation that can be observed between higher speed and more defects appearing in the finished sample. Defects, voids, etc., cause weakening of the final properties. To get rid of them, post-processing can be used. There are various methods of improving the quality of the print. There are many solutions that can be used, depending on the material and the effect that we want to achieve, i.e., whether post-processing is to improve mechanical properties, functionality, or maybe the quality of the workmanship, e.g., for decorative purposes. The basic technique includes annealing, because it improves the connections between the filament paths, thus reducing the empty spaces. This can also be combined with the impact of pressure, e.g., in a heat press. Another method is the impact of solvents, which primarily improve the quality of the surface, reducing its roughness, but can also affect the mechanical properties [54]. Both annealing and cold acetone vapour have been shown to increase the wall strength of prints, but depending on the material and the printing parameters, this can produce very different results [55].

This paper aims to study the effect of the printing speed on the strength of printed details made of PLA material, using the example of standardised shapes based on the ISO 527-1 standard [56]. Since each print under study was printed with 100% infill, we devoted special attention to how much the high printing speed affected the bonding of the inner layers of the model. was All of the prints were tested in both perpendicular X- and Y-axes to take into account the effect of the model’s alignment, i.e., the direction of the layer paths and the position of the layers relative to each other.

Fast printers that are entering the market today, such as those from Bambu Lab, make it possible to obtain speeds that were once unattainable in a regular home printing process. The mechanical properties of elements printed using these technologies have not yet been sufficiently studied. The speeds studied in the literature have rarely exceeded 200 mm/s, while in the present study, significantly higher speeds were easily achievable on new-type printers, which significantly exceed those studied so far.

This research will find applications in all areas where the new types of printers that are coming onto the market are beginning to be used, increasing the possible printing speeds to levels that were previously unattainable. Rapid prototyping, which seems to be the best application of fast printers, finds application in all areas of science and technology, from medicine to prosthetics [57] and robotics [58].

## 2. Materials and Methods

This study involved preparing eight groups of samples with varied parameters of the 3D printing process. The main objective was to investigate how the printing speed influences the strength of the parts produced. To achieve this, two key printing speeds were set: 500 mm/s and 30 mm/s, which formed the basis for the division of the tested samples. Furthermore, two supplementary variables were included in each of these groups that could potentially influence the strength properties:

- Print orientation:

Each speed setting was implemented in two variants: oriented on the X-axis and on the Y-axis.

- Use of ironing:

Printing was implemented both with and without ironing (smoothing of the top surface) enabled.

As a result, the complete set of samples consisted of eight groups (Table 1):

Each group was printed five times to accurately average the measurements obtained and minimise any deviations associated with potential instability during the sample manufacturing process. The samples subjected to tensile strength testing were prepared according to ISO 527-2 [59], which describes the tensile strength testing of polymers.

The samples were produced using 3D printing technology, a Bambu Lab A1 Mini 3D printer with a textured PEI plate (Bambu Lab, Shenzhen, China), and Bambu Lab PLA Basic green filament.

Samples’ technical data:

- Length: 170 mm;
- Width: 20 mm;
- Height: 5 mm.

Each sample was printed at 220 °C (for the first and subsequent layers), and the build plate was maintained at 65 °C throughout the printing process (Table 2).

**Figure 1:**
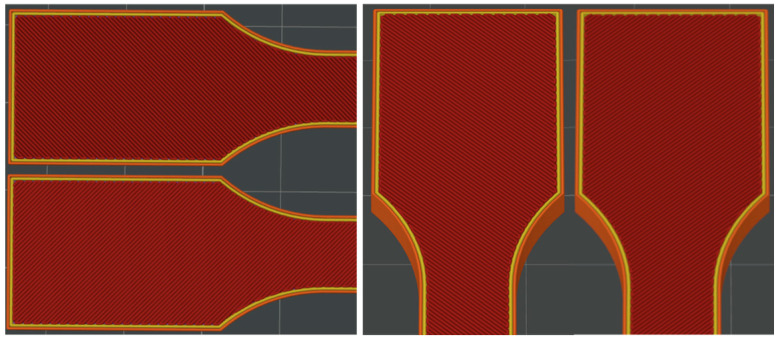
The pattern of two consecutive layers ((**left**)—the X print direction; (**right**)—the Y print direction) for each of the eight series of samples intended for the tensile strength tests. Despite the different orientation of the samples on the print bed during printing along the X- and Y-axes, the layer pattern orientation remains the same relative to the sample.

**Figure 2:**
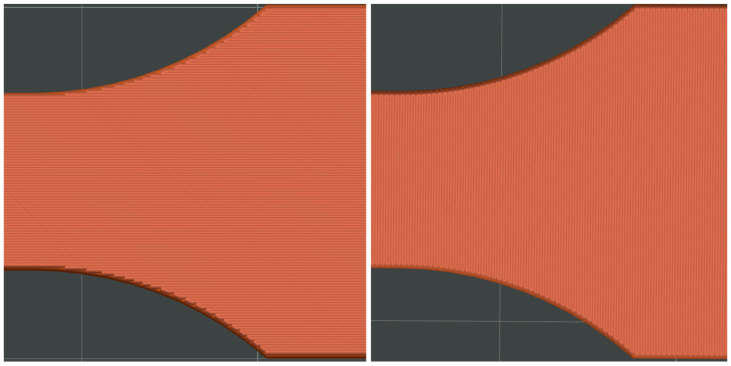
The pattern of ironing for the top layers for both the X (**left**) and Y (**right**) print directions.

**Figure 3:**
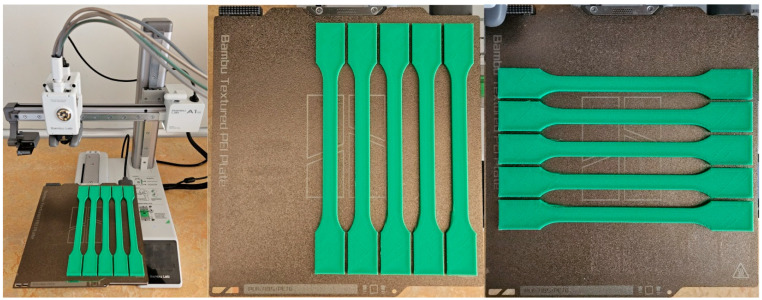
Pictures representing the printer used to produce the samples and the alignment on the platform while printing the samples for tensile strength testing.

Figure 1 illustrates the pattern of two consecutive layers for each of the eight series of samples, which were specifically designed for the tensile strength test. This figure provides a detailed view of how the layers were structured and assembled across the different sample groups. Figure 2 shows the 3D printer used for producing the samples, highlighting the alignment of the samples on the printing platform during the printing process. This image demonstrates the precise positioning required to ensure consistency and accuracy in the samples for tensile strength testing. Figure 3 captures the heat distribution on the build plate both before and during the printing process.

This image is crucial for understanding the thermal dynamics during the heating phase, which can significantly impact the final properties of the printed samples.

The ironing pattern varied depending on the orientation of the samples on the print bed. For samples printed along the X-axis, the ironing pattern was aligned longitudinally, whereas for samples printed along the Y-axis, the ironing pattern was aligned transversely.

**Figure 4:**
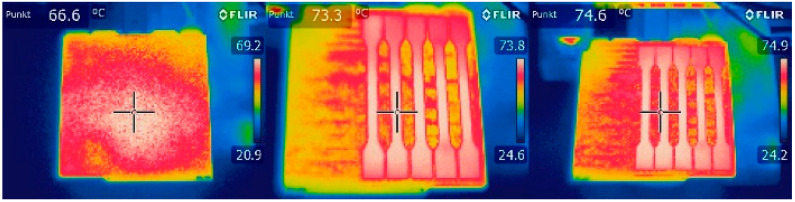
Heat distribution on the build plate during the heating process before and during printing.

The photographs below (Figure 4) were taken with a thermal imaging camera to analyse the heat distribution on the build plate during the heating process, both before and during printing.

The image on the left shows an empty build plate immediately after reaching the specified temperature value according to the printer’s thermal sensor.

The pictures in the middle and on the right display the temperature of the sample during the printing process, with the extrusion temperature set at 220 °C.

The thermal camera’s colour scale for the displayed temperatures is located on the right-hand side of each image.

**Figure 5:**
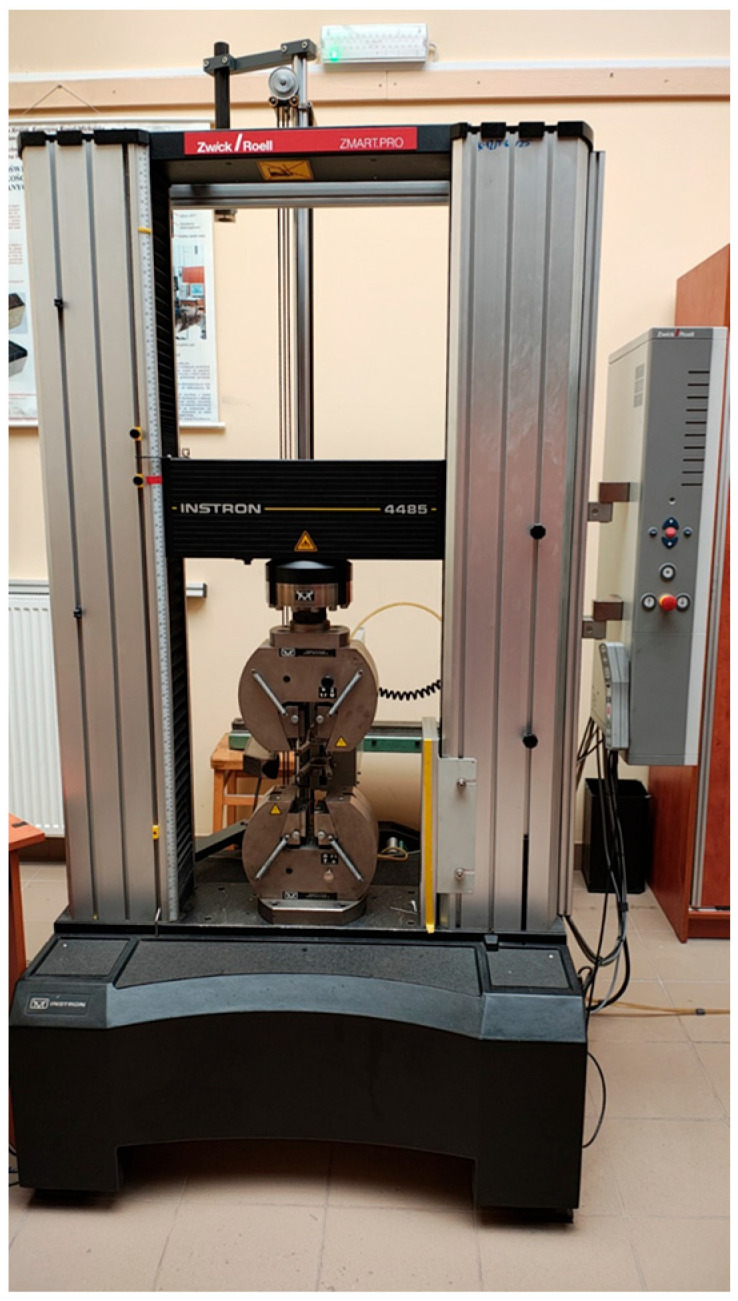
Machine used for tensile testing.

The tensile test was performed in accordance with DIN EN ISO 527-1 on an Instron 4405 (Instron, Norwood, MA, USA) universal testing machine (Figure 5) with a maximum tensile force of 200 kN. The machine was equipped with a Zwick/Roell BZ1-MM4485.IN01 (Zwick/Roell Ltd., Ulm, Germany) measuring system. The Zwick/Roell BTC-EXMACRO.M11 (Zwick/Roell Ltd., Ulm, Germany) extensometer was used for the measurements. The measurement length to determine the strain was 50 mm. The method of determining the mechanical properties was as described in the standard, and strength parameters such as Young’s modulus and conventional yield strength were determined in accordance with the standard. The testXpert II V3.2 testing machine software was used to record and process the measurements. The test conditions were defined as per the standard. The Figure 5 shows the measuring station.

To further investigate the mechanical properties, a three-point bending test was conducted in accordance with ISO 178 [60], and an impact strength test was performed in accordance with ISO 179 [61]. Both tests utilised rectangular prism-shaped specimens with dimensions of 80 × 10 × 4 mm.

Statistical analyses were conducted using Origin Pro 2020 SR1 v.9.7.0.188 (OriginLab Corp., Northampton, MA, USA). A three-factor analysis of variance (ANOVA) was applied with a significance level of α = 0.05 to determine statistical significance. This analysis allowed us to rigorously evaluate the influence of the tested parameters on the mechanical properties under study.

## 3. Results

**Figure 6:**
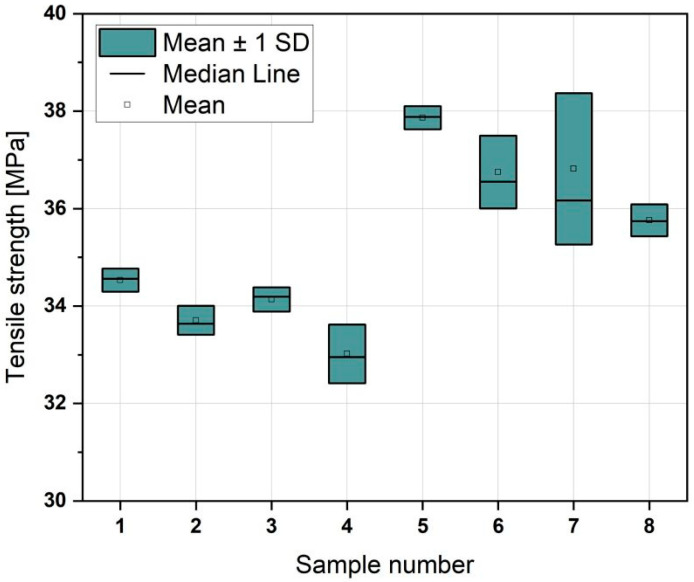
Tensile strength of samples printed at different speeds.

The results presented in Figure 6 show that samples printed along the Y-axis exhibited greater tensile strength. The Y-axis-printed samples achieved higher average stress values than the corresponding samples printed along the X-axis. The chart also indicates that the choice of axis is considerably more critical than the other parameters that we studied, such as print speed or the presence of ironing.

Upon analysing the remaining parameters, it becomes evident that samples printed at reduced speeds exhibit marginally superior tensile strength compared to those printed at higher speeds (Sample 1 demonstrates greater strength than Sample 3; similarly, Sample 2 exhibits greater strength than Sample 4). Furthermore, samples that underwent ironing after the printing process exhibited marginally elevated tensile strength values, with Sample 1 demonstrating superior results relative to Sample 2, and Sample 3 displaying greater strength than Sample 4. This correlation was more pronounced in samples printed along the X-axis, while being somewhat less evident in those printed along the Y-axis. Additionally, for samples printed at a speed of 500 mm/s, there existed a higher degree of variability in the strength results when compared to those printed at 30 mm/s.

**Figure 7:**
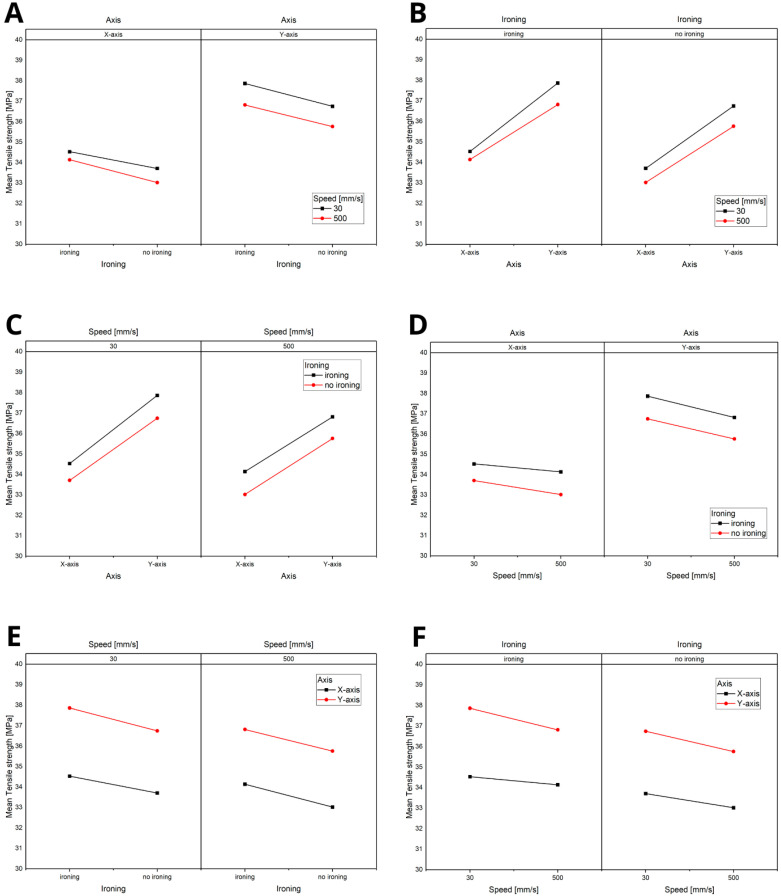
Title: Statistical Analysis of the Significance of Parameters in the Tensile Strength Test: (**A**) analysis of the influence of speed on the variables: printing axis and the application of the ironing option, (**B**) analysis of the influence of speed on the variables: application of the ironing option and printing axis, (**C**) analysis of the influence of ironing on the variables: speed and printing axis, (**D**) analysis of the influence of ironing on the variables: printing axis and printing speed, (**E**) analysis of the influence of the axis on the variables: printing speed and ironing, (**F**) analysis of the influence of the axis on the variables: ironing and printing speed.

Based on the significance analysis (Figure 7), it was demonstrated that the most influential factor is the axis, followed by ironing and, finally, speed. As shown in the analysis, interactions between the parameters were not present, with all *p*-values being greater than 0.05. This indicates that each parameter independently affects the material properties without significant interactive effects.

**Figure 8:**
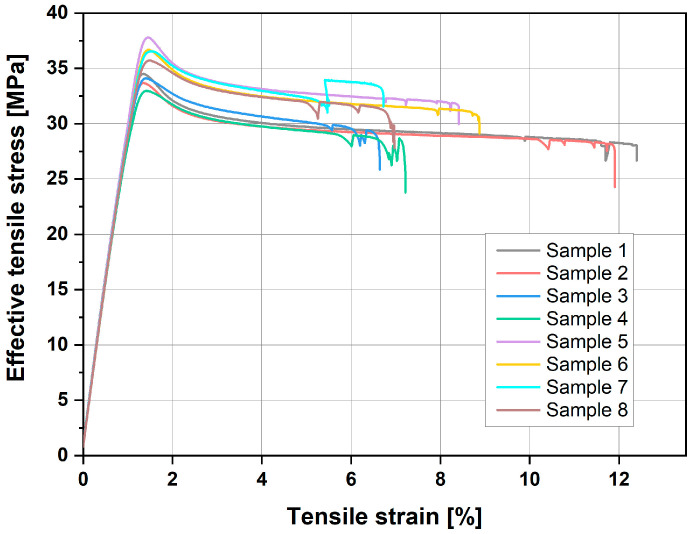
Stress–strain curves of samples printed at different speeds.

Figure 8 presents the stress–strain curves of samples printed at different speeds. This graph compares the mechanical behaviour of the samples under tensile stress, showing how varying printing speeds influence their strength and elasticity. By analysing these curves, we can gain insight into the material properties and performance of the samples produced at different print speeds.

The trends described above are visible at the peak of the stress–strain curve based on the average values. The data suggest that higher tensile values are obtained for samples printed along the Y-axis (Samples 5 to 8) than for those printed along the X-axis (Samples 1 to 4). The curve also demonstrates that greater elongation is observed in samples printed at 30 mm/s (Samples 1 and 2, as well as 5 and 6). There is also a noticeable difference between the axes, as samples printed along the X-axis elongated more before breaking than those printed along the Y-axis. Samples printed at 500 mm/s showed no significant differentiation in terms of elongation based on the axis. Similarly, no relationship between ironing and elongation could be established.

**Figure 9:**
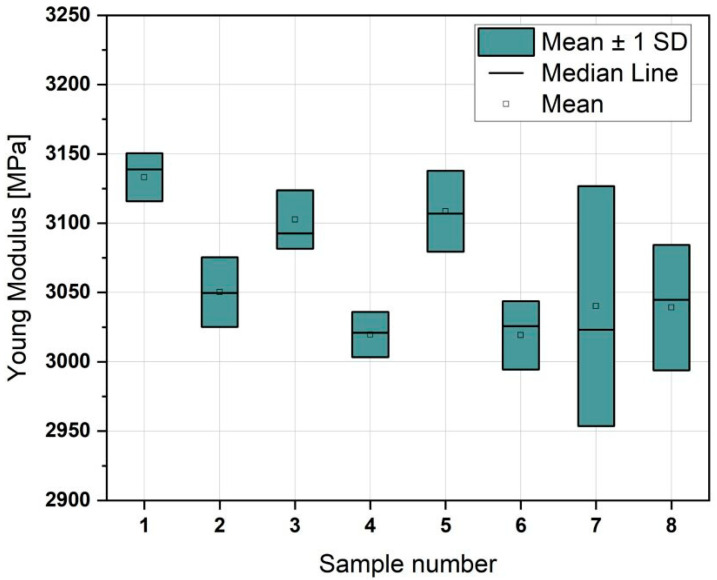
Young’s modulus of samples printed at different speeds.

The situation differs when considering Young’s modulus (Figure 9).

Here, the values are within a similar range, suggesting that the modulus is less dependent on the printing parameters and primarily results from the material used. When plotting the results, it is evident that the only parameter that appears to influence Young’s modulus value is the use of ironing. Samples subjected to ironing (Samples 1, 3, and 5) generally show higher modulus values than those without ironing (Samples 2, 4, and 6). This pattern is disrupted only by the results obtained for Samples 7 and 8, which show greater variability in results, preventing a clear conclusion.

**Figure 10:**
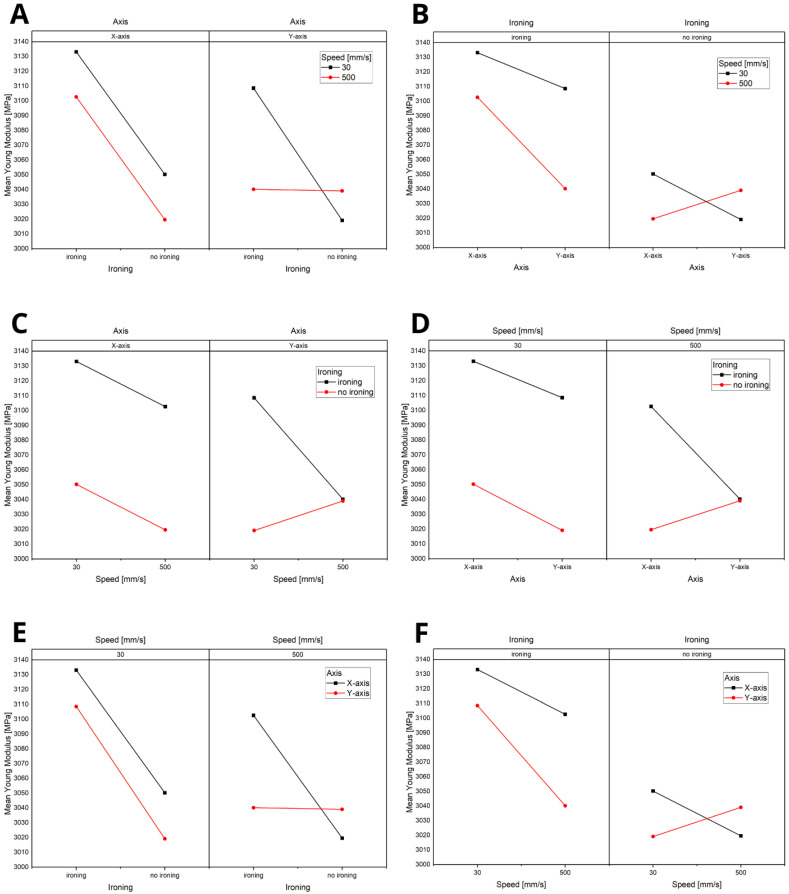
Title: Statistical Analysis of the Significance of Parameters in the Young’s Modulus Test: (**A**) analysis of the influence of speed on the variables: printing axis and the application of the ironing option, (**B**) analysis of the influence of speed on the variables: application of the ironing option and printing axis, (**C**) analysis of the influence of ironing on the variables: printing axis and printing speed, (**D**) analysis of the influence of ironing on the variables: speed and printing axis, (**E**) analysis of the influence of the axis on the variables: printing speed and ironing, (**F**) analysis of the influence of the axis on the variables: ironing and printing speed.

The statistical analysis (Figure 10) revealed that ironing has the most significant impact, followed by speed. The axis is practically insignificant in influencing the outcomes. Moreover, interactions between the parameters are nonexistent, with all *p*-values exceeding 0.05. This suggests that each parameter functions independently, without notable interactive effects on the material properties.

The photos in the table show the topography of sample fractures made at 100-fold magnification. It can be observed that the fractures of samples printed at a lower speed (Samples 1, 2, 5, and 6) are more uniform, with visible filament paths. The differences between the extreme points are usually below 1000 µm. On the other hand, the samples printed at higher speeds (Samples 3, 4, 7, and 8) have more irregular, jagged fractures, with significant differences between the extreme points, reaching over 2000 µm. Such a large surface unevenness means that filament paths are not visible in the profile.

Table 3 displays fracture images, showcasing each group’s most distinctive samples. These images highlight the different fracture patterns observed in the samples, visually representing their mechanical failure. By analysing these fractures, we can better understand the material behaviour and the impact of various printing conditions on the structural integrity of the samples.

SEM observations were conducted on two samples—specifically, Samples 1 and 8—to gain a more detailed view of the filament paths and emerging defects. The images obtained from this examination are compiled in Table 4.

The analysis revealed no significant differences in the filament paths or the visible defects between the samples. However, distinct fracture patterns were observed. Sample 1, which was printed at a lower speed, exhibited a more brittle fracture. In contrast, samples printed at higher speeds and in a different orientation demonstrated a more ductile fracture.

The KEYENCE VHX-950F (Keyence Corporation, Osaka, Japan) is a high-performance optical microscope equipped with a 1/1.8-inch CMOS image sensor, offering a resolution of 1600 × 1200 virtual pixels. It features both automatic and manual settings for focus, white balance, and shutter speed, with up to 50 full-frame images per second. This microscope was used to analyse the fractures of the samples shown in Table 5.

Samples were placed vertically under the microscope, with the cross-section parallel to the optics. The topography scan was performed from the centre of the sample whenever possible.

**Figure 11:**
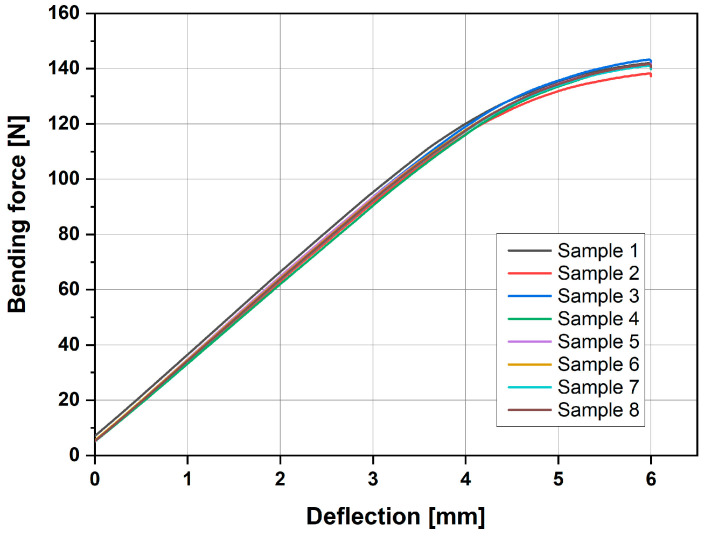
Graph representing the bending force of samples printed at different speeds.

In addition to the tensile and surface analyses, three-point bending tests were conducted to further assess the mechanical properties of the materials. The results of these tests are illustrated in Figure 11.

Upon analysis, we found that there were no significant differences among the different series of samples, with each series consisting of five specimens. Furthermore, none of the parameters that we investigated had a noticeable impact on the bending performance. The variations observed in the bending behaviour were statistically insignificant, reinforcing the conclusion that the tested parameters do not substantially influence the material’s response under three-point bending conditions. This suggests a level of consistency and robustness in the material’s bending characteristics across the different sample sets.

**Figure 12:**
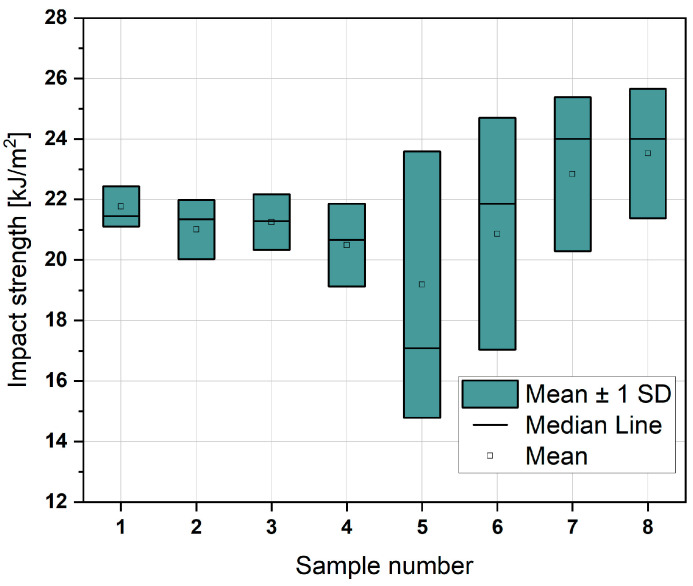
Graph representing impact strength according to sample series number.

Additionally, an impact strength test was conducted, and the results are compiled in Figure 12.

The Y-axis contributed to an increased dispersion of the results, resulting in a larger standard deviation. This indicates greater variability in the data along this axis. However, despite this variability, the results overlap and remain within the bounds of the standard deviation. This consistency suggests that the outcomes, while variable, align closely with one another and fall within expected limits, maintaining reliability across the dataset.

**Figure 13:**
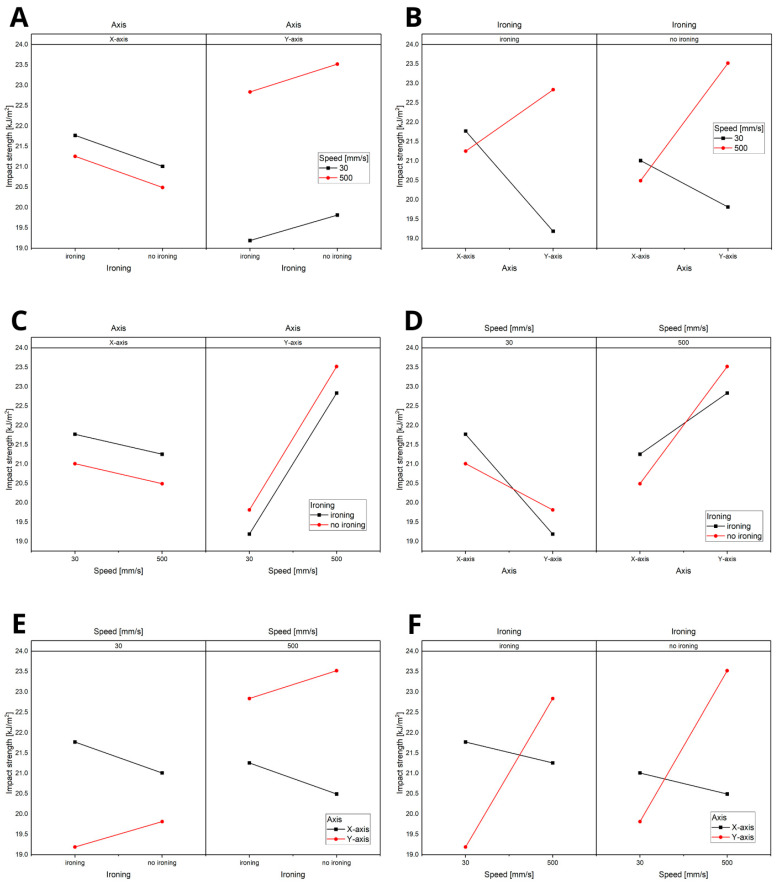
Statistical Analysis of the Significance of Parameters in the Impact Strength Test: (**A**) analysis of the influence of speed on the variables: printing axis and the application of the ironing option, (**B**) analysis of the influence of speed on the variables: application of the ironing option and printing axis, (**C**) analysis of the influence of ironing on the variables: printing axis and printing speed, (**D**) analysis of the influence of ironing on the variables: speed and printing axis, (**E**) analysis of the influence of the axis on the variables: printing speed and ironing, (**F**) analysis of the influence of the axis on the variables: ironing and printing speed.

The statistical analysis, summarised in Figure 13 (ranging from A to F), revealed that the axis and speed are the key influencing parameters. Notably, speed plays a significant role only when considering the Y-axis. This finding is related to the direction of the hammer’s impact during the impact strength test, emphasising the importance of orientation and velocity in determining the material’s dynamic response.

## 4. Discussion

This study aimed to determine the impact of 3D printing speed on the mechanical strength of parts produced using this technology. The tests were based on the ISO 527-1 standard, where the samples were subjected to a tensile strength test. The results indicate that the orientation of the samples on the build platform also significantly impacts their strength properties.

No visible differences were found in the visual assessment of the surface quality of the samples (for samples without ironing enabled (Samples 2, 4, 6, and 8), which smooths the upper surface of the sample), suggesting that increasing the printing speed from 30 mm/s to 500 mm/s, with an appropriately selected material capable of effective melting at this extrusion speed, does not affect the quality of the printed parts.

Thermal camera images were used to support the analysis of the fracture points of the sample series, aiming to detect any irregularities in the heat distribution on the build plate, which could potentially explain the non-reproducible fracture points during the tensile strength testing. Analysis of the thermal camera images revealed no significant deviations in the regularity of heat dissipation on the build plate during heating, excluding this factor as a determinant of the lack of reproducibility of the sample fracture points.

Surface fracture analysis showed that samples printed at a speed of 30 mm/s had a more even fracture surface than those printed at 500 mm/s. A trend was also observed where the use of the ironing function improved the consistency of the fracture surface regardless of the speed used, resulting in a flatter break. SEM microscope images revealed that samples printed at lower speeds tended to display more brittle fracture characteristics, whereas those printed at higher speeds showed a more ductile fracture nature. This observation suggests a potential relationship where printing speed influences the type of fracture, which could impact the material performance and mechanical properties.

Based on the collected data, we can conclude that, when using high-speed PLA in our case, Bambu PLA Basic (Shenzhen Tuozhu Technology Co., Ltd., Shenzhen, China) —which is primarily used for prototypes and decorative elements such as figurines, decorative vessels, or other components not designed to operate under heavy loads at room temperature—manufacturing parts at higher speeds (such as at 500 mm/s in this study) reduces the mechanical strength of the printed elements but allows for maintaining appropriate print quality.

Considering the application of this material, however, it can be concluded that the difference in mechanical strength between prints made at 500 mm/s and 30 mm/s is minimal. This brings significant time savings, which can be crucial in prototyping and developing production processes that use 3D printing. It is also more economical due to shorter energy consumption.

However, it should be noted that not all types of PLA can adequately melt at such extrusion speeds, which could result in gaps in the structure or other defects that compromise the strength and aesthetics of the parts. The analysis of the results indicated that increasing the printing speed reduces the strength properties of the produced samples. Samples printed at 500 mm/s (Samples 3, 4, 7, and 8) exhibited lower strength than samples printed at 30 mm/s (Samples 1, 2, 5, and 6).

The tensile strength analysis indicated that samples printed along the Y-axis perform better than those printed along the X-axis. This observation suggests that the printing axis is dominant in determining the mechanical properties, overshadowing the impact of other factors, such as print speed and ironing. This was confirmed by the statistical significance analysis of the parameters. Specifically, printing at lower speeds (30 mm/s) (Samples 1, 2, 5, and 6) enhances tensile strength, and ironing at the end of the print also results in slightly improved strength. However, this effect is less consistent for Y-axis prints. The tensile strength values for PLA, ranging from 25 to 40 MPa, are consistent with those reported in the literature [34,54].

The results regarding Young’s modulus indicate that this property is primarily influenced by the material and not significantly by the print parameters. Ironing seems to increase the modulus, although this relationship is disrupted for high-speed prints, where the results showed greater variability. The influence of ironing was further confirmed by the statistical significance analysis, which indicated that this parameter has the most substantial impact on Young’s modulus.

It can be observed that the ironing directions specified in Figure 2 showed differences in tensile strength when comparing samples printed at the same speed but oriented along opposite axes, with and without ironing. When comparing Samples 3 (ironing) and 4 (no ironing) to 7 (ironing) and 8 (no ironing), we can observe a higher increase in tensile strength for samples with longitudinal ironing compared to those without ironing, relative to the increase in tensile strength for samples with transverse ironing compared to those without ironing. However, this was not confirmed when comparing the other two sets: Samples 1 and 2 for longitudinal ironing, and Samples 5 and 6 for transverse ironing. In this case, a higher increase in tensile strength was observed for the samples with transverse ironing compared to those without ironing. This suggests that the ironing direction is a difficult variable to determine in terms of whether it improves the mechanical properties of printed parts. Studies on the impact of the ironing direction on mechanical strength could provide valuable insights into whether process parameters such as flow rate and speed are more significant than the orientation of the ironing. At this point, it is more of a cosmetic technique used to improve the quality of the upper surface of the print.

The strong influence of the print axis on tensile strength can be attributed to the alignment of the material layers and the orientation of the printed fibres, which may provide more or less resistance depending on the direction. This seems to confirm the conclusion from the author’s study [53] that this is the most important parameter for the final strength. The Y-axis orientation seems to offer better structural integrity under tension.

A three-point bending test revealed negligibly small differences among the various sample series. It can be assumed that the quality of the samples, including their porosity and the presence of defects, was not significantly influenced by the parameters studied in this work.

In impact testing, the orientation of the samples significantly affected the repeatability of the results. Samples printed along the X-axis exhibited a lower standard deviation compared to those printed along the Y-axis. Furthermore, statistical analysis indicated that the combination of these two parameters is critical for the final impact strength. The printing speed proved to be particularly significant when the samples were printed along the Y-axis. These findings are likely correlated with the direction of the hammer impact in the impact testing, emphasising the importance of carefully planning the specimens’ orientation during the printing process if they are expected to undergo loading.

The effect of print speed on strength was consistent with previous research, suggesting that lower speeds allow for more precise layer bonding and fewer inconsistencies in the material structure, leading to stronger prints. However, higher-speed prints exhibit more unpredictable failure behaviour, which may indicate that the faster extrusion rate and cooling process lead to less consistent interlayer bonding.

Ironing, which smooths the surface at the end of the print, enhances the overall mechanical properties, particularly the tensile strength. However, this effect is not always uniform across all sample orientations. Its influence on Young’s modulus is more evident, showing higher values for samples subjected to ironing, likely due to the more compact surface structure created by the ironing process.

In conclusion, while the printing axis and speed play significant roles in determining the mechanical properties, ironing notably impacts specific properties, such as tensile strength and modulus, albeit with some variability depending on the print orientation and speed. Further investigation with more controlled variables could help clarify these trends and provide more precise guidelines for optimising 3D printing processes for desired mechanical outcomes.

## 5. Conclusions

- Increasing the print speed from 30 mm/s to 500 mm/s decreases the mechanical strength of the printed parts, but the differences are not significant. It also does not significantly affect the surface quality, provided that the material (high-speed PLA) is properly selected. High-speed PLA (e.g., Bambu PLA Basic) is suitable for parts used in prototypes and decorative elements but is not intended for high-load applications. Printing at higher speeds reduces strength but saves time and energy.
- The ironing function (smoothing the surface) improves the consistency of the fracture surface, regardless of print speed. Using this function positively impacts the tensile strength, resulting in a flatter fracture surface.
- The orientation of the samples on the build plate significantly impacted the final results. Although the heat distribution on the plate was uniform, differences in sample orientation affected the quality and mechanical strength of the printed parts, particularly in terms of material layering and structure. Parts printed along the Y-axis exhibited better tensile strength than those printed along the X-axis, indicating better material layer alignment and providing superior structural integrity under stress.
- Young’s modulus is primarily influenced by the material rather than the print parameters. However, ironing increases this property, and higher print speeds show more variability, indicating less stability in the structure when printed faster.
- From the statistical significance analysis, it can be inferred that the axis has the greatest impact on tensile strength, while ironing, which pertains to surface quality, most significantly affects Young’s modulus.
- None of the parameters investigated in the study had an impact on the bending test results.

The statistical analysis revealed that, in the case of impact strength testing, the most significant parameter is orientation, while for the Y-axis, speed is also a critical factor.

## Figures and Tables

**Table 1:** Full description of the samples used in the experimental tensile tests.

Sample Number	Parameters
1	Speed 30 mm/s, X-axis, with ironing
2	Speed 30 mm/s, X-axis, without ironing
3	Speed 500 mm/s, X-axis, with ironing
4	Speed 500 mm/s, X-axis, without ironing
5	Speed 30 mm/s, Y-axis, with ironing
6	Speed 30 mm/s, Y-axis, without ironing
7	Speed 500 mm/s, Y-axis, with ironing
8	Speed 500 mm/s, Y-axis, without ironing

**Table 2:** Printing parameters for the samples representing high-speed printing (500 mm/s) and low-speed printing (30 mm/s).

Printing Parameter	Value (500 mm/s)	Value (30 mm/s)
Layer height	0.2 mm	0.2 mm
Initial layer height	0.2 mm	0.2 mm
Line width	Basic settings	Basic setting
Wall printing order	Outer/inner	Outer/inner
Wall loops	2	2
Top surface pattern	Rectilinear	Rectilinear
Top shell layers	4	4
Bottom surface pattern	Rectilinear	Rectilinear
Bottom shell layers	4	4
Sparse infill density	100%	100%
Sparse infill pattern	Rectilinear	Rectilinear
Infill/wall overlap	20%	20%
Initial layer speed	50 mm/s	30 mm/s
Initial layer infill	105 mm/s	30 mm/s
Outer wall	500 mm/s	30 mm/s
Inner wall	500 mm/s	30 mm/s
Sparse infill	500 mm/s	30 mm/s
Internal solid infill	500 mm/s	30 mm/s
Top surface	500 mm/s	30 mm/s
Normal printing acceleration	10,000 mm/s^2^	6000 mm/s^2^

**Table 3:** Images of fractures representing the most distinctive samples in the group.

Sample Group Number	Images of Fractures Representing the Most Distinctive Samples in the Group
1	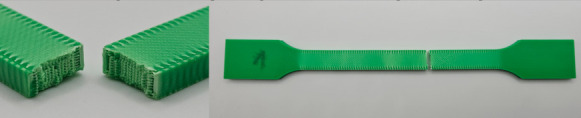
2	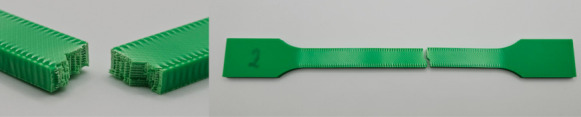
3	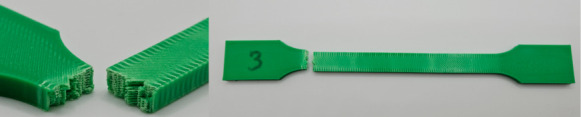
4	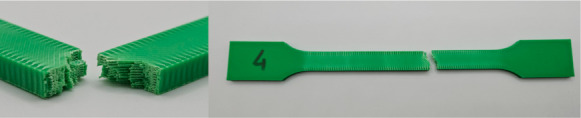
5	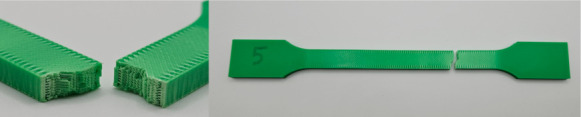
6	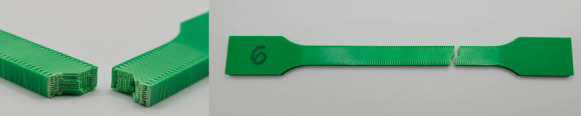
7	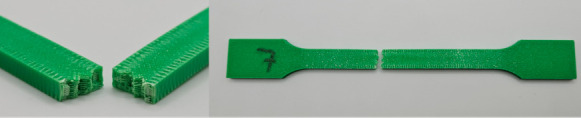
8	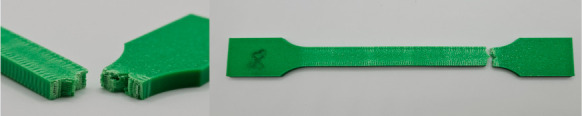

**Table 4:** Images of fractures representing the most distinctive sample series.

Sample Group Number	Images of Fractures Representing the Most Distinctive Samples in the Group, Taken on an SEM Microscope
1	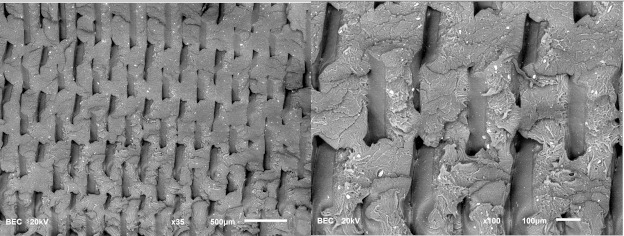
8	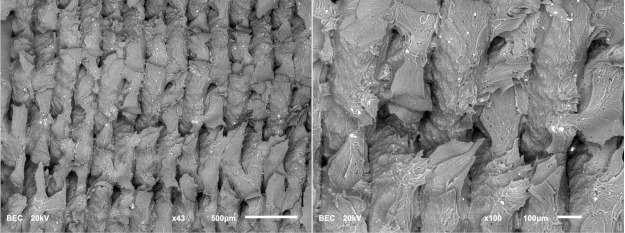

**Table 5:** Optical microscopy images of fractured surfaces represent the group’s most distinctive samples.

Sample Group Number	Images of Fractures Representing the Most Distinctive Samples in the Group
1	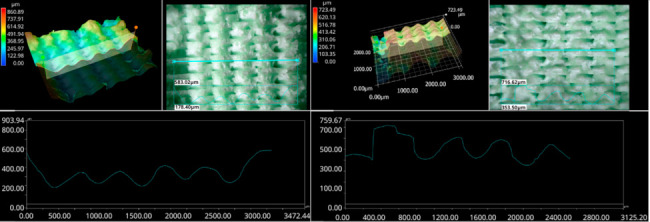
2	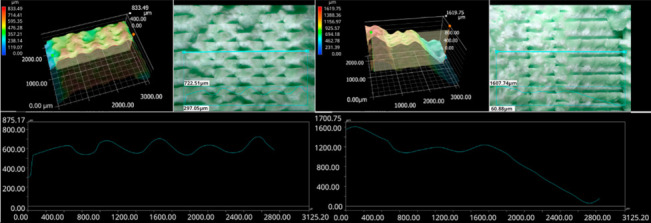
3	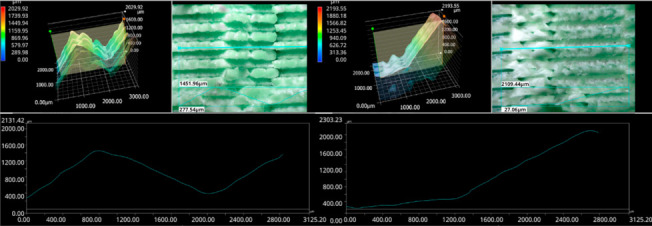
4	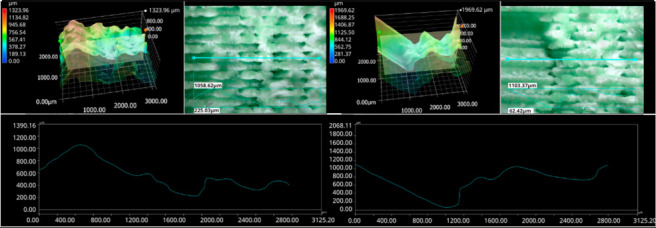
5	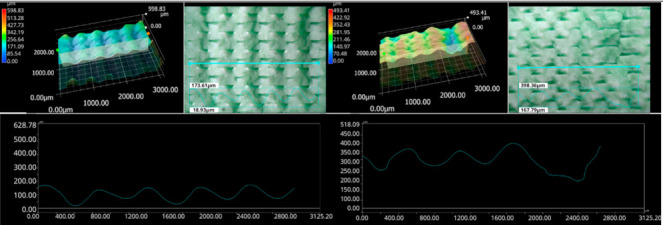
6	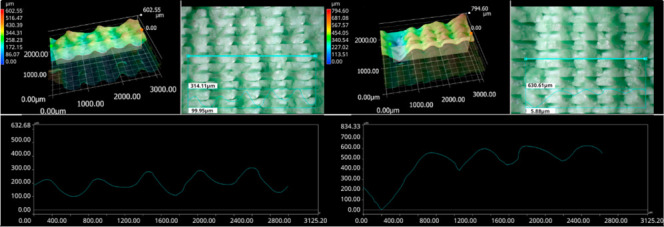
7	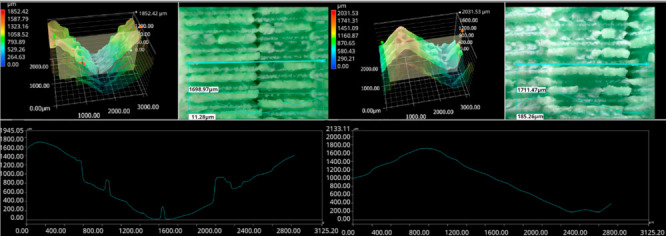
8	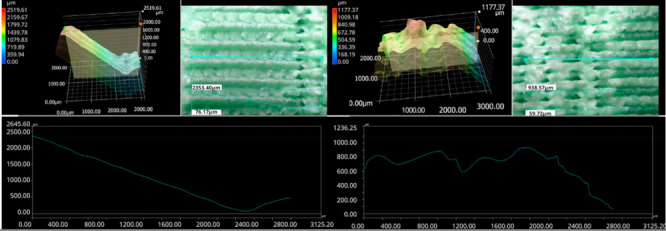

## Data Availability

The original contributions presented in this study are included in the article. Further inquiries can be directed to the corresponding author.
